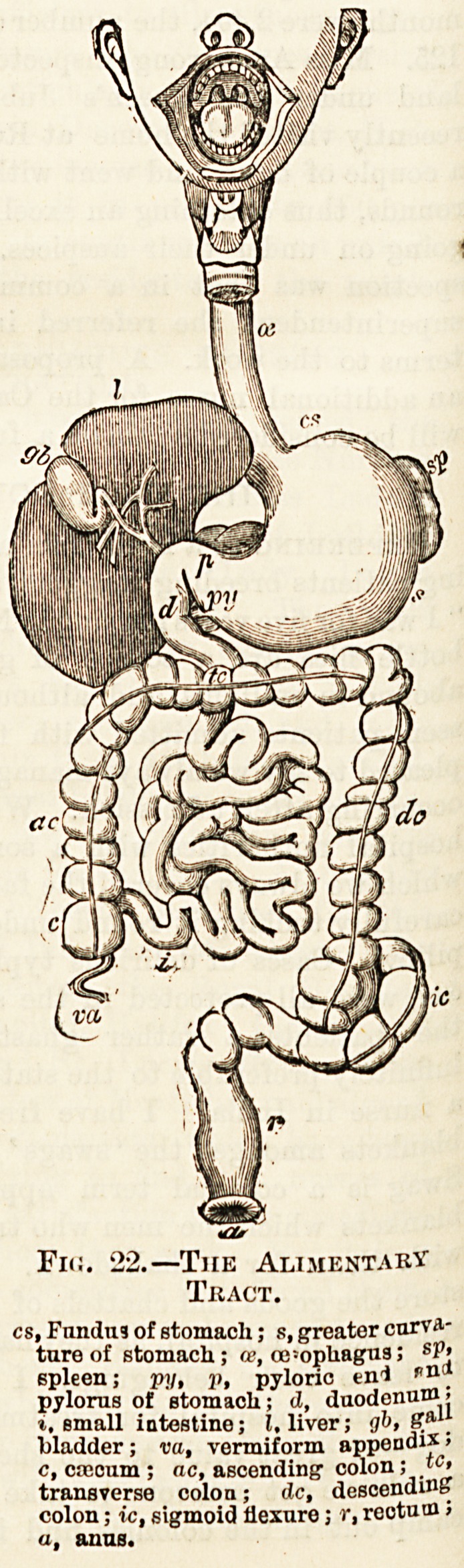# "The Hospital" Nursing Mirror

**Published:** 1900-08-04

**Authors:** 


### "The Hospital" Nursing Mirror.

Being the Nursing Section of "The Hospital."

[Contributions for this Section of "The Hospital" should be addressed to the Editor, The Hospital, 28 & 29, Southampton Street, Strand, London, W.C., and should have the word "Nursing" plainly written in left-hand top corner of the envelope.]

### Notes on News from the Nursing World.

### ENGLISH MATRONS IN FRANCE.

We have been asked to find a fully-qualified matron for an important hospital in France, who has a good knowledge of French, the latter qualification being essential. We shall be glad to have the names of any ladies who may be qualified for the post. Preference will be given to those trained and recommended by the lady superintendent of one of the larger hospitals. All applications should be made by letter addressed to the Editor, The Lodge, Porchester Square, London, W., marked "Foreign" in the left hand top corner.

### MISS FLORENCE NIGHTINGALE AND THE INDIAN FAMINE.

It is announced that Miss Florence Nightingale has forwarded to the *Manchester Guardian* "Indian Famine Fund" a cheque for £100. The contribution was enclosed "with Florence Nightingale's keenest sympathy for the sufferers and admiration of those who are doing so much for them with heart and hand." This generous gift shows how closely Miss Nightingale continues, in her retirement, to follow the events of the day, and is one more evidence of the sympathy which she feels for suffering humanity in all parts of the world.

### A FURTHER DISPATCH OF NURSES TO THE FRONT.

The Secretary of State for War informs us that the following 20 members of the Army Nursing Service Reserve will embark for South Africa on Saturday: Miss A. E. André, Miss H. Anderson, Miss M. Barton, Miss M. B. Bennett, Miss M. Clements, Miss A. French, Miss J. Halliday, Miss M. Henderson, Miss A. B. Hill, Miss M. Hodges, Miss M. E. Howell, Miss E. Johnson, Miss A. S. Liell, Miss F. Puddicombe, Miss E. Seddon-Smith, Miss A. E. Smith, Miss M. M. Tunley, Miss A. M. B. Usher-Somers, Miss L. L. Watts, and Miss A. M. Wright. Miss Margaret Henderson was trained at the Cottage Hospital, Hawick, and has been engaged in private nursing at the institute, Sunderland, since May, 1897. Miss Amy Bland Hill was probationer at the London Hospital, Sussex County Hospital, the General Infirmary (Birmingham), and the New York Hospital (New York), in succession. She was afterwards charge nurse at the last-named institution; and since 1896 she has been Queen's District Nurse at Anghrim, county Wicklow. Miss Mildred Hodges was trained at the Royal Infirmary, Edinburgh, where she was afterwards night nurse. She has since been district probationer, Queen Victoria Jubilee Institute for Nurses, Central Home, Bloomsbury; army district nurse at Aldershot; and nurse at the General Hospital, Birmingham. From 1897 she has been attached to the Nurses' Co-operation.

### A NURSE ON NIGHT DUTY AT MARITZBURG.

A striking proof of the inadequate supply of nurses in South Africa at one period of the war is afforded by a sister at Maritzburg, who was in charge of 500 beds at night. She made the following statement to a correspondent of the *Daily News* in the town:—

"Two wards containing sixty beds, with no orderly, one case of acute bronchitis and several helpless surgical patients in splints. Two wards containing fifty beds, no orderly, dysentery and helpless surgical cases (splints). Two wards of fifty beds, containing fever, dysentery, suspected enteric, and helpless surgical cases, no orderly. Two more wards containing same as above, no orderly. Diets and extras drawn in morning, fever cases admitted in afternoon. Supplied with milk as diet, no supply of soda water being allowed in wards except what is ordered daily for those already in. Orderlies on night duty go on duty seven p.m.; off duty at seven a.m., no food being supplied for that duty of twelve hours. Cases of acute dysentery and enteric have been known to get out of bed, either because there was no orderly on or because the orderly was asleep and could not be waked, the reason being given that the orderly had been on duty the whole of the previous day. Orderlies are changed sometimes daily, therefore lose heart and take no interest in their work or patients."

The members of the South African Hospitals Commission, who start on Saturday for the Cape, will doubtless take care that allegations of this character are inquired into, as also the further assertion that "nursing sisters were not allowed to requisition for anything, on pain of dismissal."

### "TOMMY" ON CURRENT AFFAIRS.

"What do you think of the hospital scandals?" said the sister of a ward to some of her patients at a military hospital. "We're beginning to think its the volunteers as made the complaints." "And the C.I.V.'s," chimed in another; "they thought they were coming out to a fine picnic; no Tommy has made any complaints." "But," objected the sister, drawing out fuller comment, "if the dead and sick lay side by side, and enteric cases had mud, water, and a blanket to lie on, it was time someone drew attention to the subject. If Mr. Burdett Coutts saw these things it was right for him to speak." "I don't think he noticed till someone put him up to it," said Private ——," and, voicing the feeling of the ward, he added, "if Tommy doesn't complain I don't see why anyone else need." The sister left the ward proud that that was the stuff of which British soldiers are made, yet more persuaded than ever that brave, silent endurance should meet recognition by remedy of avoidable evils.

### BOER PATIENTS.

It will be seen from a report in another column, that Miss Pope, who was in Kimberley throughout the siege, and assisted in nursing more than 150 Boers who were sent into the town after the flight at Paardeberg, speaks highly of the Boers as patients. Unlike Madame Alice Bron, she did not find them at all difficult to manage. They were Cronje's party, and included several notable men. Miss Pope says that when Lord Methuen sent them in, he expressed a desire that they should be treated just as if they were British soldiers, and that they had every possible care and attention. Referring to her experiences during the siege, she confessed that if she had known what was before her when she went out, she does not think that she could have faced it. Most of us understand that feeling, and are correspondingly grateful that the future is hidden from us.

### THE COLONIAL NURSES FOR SOUTH AFRICA.

The Quartermaster-General informs us that, in addition to the nursing staff of the transport "Avoca," which left for South Africa last month, there were on board the vessel nine Colonial nurses, namely, Mrs. Pringle, and the Misses Bradbury, Borlase, Chaney, Charles, Dawson, Holgate, Moore, and Paterson. The staff was as follows: Misses Yeatman, Nicholson, Anderson, Robertson, Makepeace, and Grewer.

### THE NURSES OF PRESTON ROYAL INFIRMARY.

On Saturday evening the prizes awarded as the result of the final examination were presented to the successful nurses by the chairman of the Preston Board of Guardians, Mr. W. P. Park, in the Board Room. The first prize was won by Miss Hilda Cavanagh, who obtained 169 marks; the second by Miss Edith Hurst, who obtained 162 marks; and the third by Miss Amy Landon, who obtained 155 marks. The prizes were medical books, and were the gift of the chairman. The practical examination was conducted by Dr. Falconer, the senior house surgeon, and the papers were corrected by Dr. Garner, one of the honorary medical staff.

### "TOO GOOD FOR A WORKHOUSE."

According to a report in the *Shrewsbury Chronicle*, the Atcham Board of Guardians have had a superintendent nurse who was too good for a workhouse. The Chairman announced that Miss Preece, who was only recently selected for the position of superintendent nurse, had resigned. Both the chairman and the master of the workhouse said that Miss Preece was "too good for the place." The statement was made seriously, not ironically; and the master said "they could not keep that class of nurse; they would not put up with paupers." We hope, however, that he did injustice to Miss Preece, for all nurses worthy of their high calling will nurse sick paupers as readily and as devotedly as any other class of suffering humanity. It is, as a rule, not the patients, but the conditions of nursing in workhouses under boards of guardians which are objected to by nurses and cause the difficulties in obtaining and in keeping them. On this subject we have pleasure in calling the attention of our readers to the report of Inspector Fleming of the Local Government Board, which we give in another part of the paper.

### A COMPLIMENT TO SLOUGH NURSES.

The General Manager of the Great Western Railway has informed Miss Herschell, the honorary secretary of the Slough Nursing Institution, that the directors of his company have decided to make a donation of ten guineas to the institution, "in recognition of the very valuable assistance rendered by its two trained nurses to the injured after the recent terrible disaster at Slough." This is a graceful and suitable mode of acknowledging the services of the nurses, and we are glad that the directors of the Great Western Railway have thus shown their sense of the importance of the help afforded.

### MINING COMPANIES AND NURSING ASSOCIATIONS.

The precedent set by the West Kitty Mining Company in deciding that two guineas be for the future annually subscribed to the St. Agnes District Nursing Association is one which it is hoped may be practically approved and recognised in Cornwall and by other mining companies of other counties. On inquiry we find that this is purely a voluntary subscription, the result of the strict and kindly attention of nurse to sick persons employed at West Kitty Mine. Although everything possible may be done by mining companies for the security of workmen, immunity from accident cannot be ensured, and how great the boon both to medical man and to miner to be able to secure the trained assistance of a district nurse can scarcely be imagined, particularly in districts some miles from hospital, where, owing to scattered population, only one doctor is in residence. Nor is it only in cases of accident that the nurse is found valuable. It is astonishing in how many instances her presence is a comfort to the afflicted. Indeed, the introduction of nursing associations into Cornwall has been one of the greatest blessings ever conferred in this county, alike to the very poor, and also to others of the working classes whose earnings are not sufficient to allow them to procure trained nursing assistance. The St. Agnes Nursing Association is this year particularly fortunate in one of its chief friends, Sir Edwin Durning Lawrence, M.P., having invested towards the income of the association a sufficient sum to secure it in perpetuity £50 per annum, which will ensure its continuance as an unfailing source of relief to the residents of the neighbourhood.

### THE MEATH WORKHOUSE NURSING ASSOCIATION.

The following ladies have recently joined the Executive Committee of the Meath Workhouse Nursing Association: Her Serene Highness Princess Ludwig of Loewenstein; Constance, Marchioness of Lothian; the Countess of Stafford, the Lady Louisa Egerton, and the Hon. Mrs. Egerton. The work of the association, which has been steadily growing for the last five years, has thus received a fresh impulse. More probationers are required, and more vacancies in which to train them are being provided. At the present time 62 nurses belonging to the association have obtained poor law appointments, and 36 probationers are in training for the work. Among the institutions which have hitherto been used for this purpose are the Crumpsal Infirmary, Manchester; the General Infirmary, Worcester; the Royal Hospital, Sheffield; St. Luke's Hospital, Halifax; the General Hospital, Cheltenham; and St. Lucy's Home, Gloucester. Hitherto, the Countess of Meath has paid almost all the expenses of the organisation, which have been considerable. It is gratifying to find that nurses of the association have given satisfaction to the guardians and the matrons under whom they work. But it is a matter for regret that the committee have found themselves unable to supply nurses to several county boards which have applied for them. Happily the evidence before them of the need which exists for the work of their association is on the increase and leads them to hope that support for it will also increase. The hon. secretary, Miss Annie Lee, will be pleased to give or receive any information on the subject at the office, 51, Upper Baker Street, N.W., any afternoon, between two and four, Saturdays excepted.

### A RECENT APPOINTMENT BY THE BIRKENHEAD GUARDIANS.

Communications on this subject continue to reach us, and one of our numerous correspondents expresses surprise that we should think there is nothing more to be said in the matter. Writing on behalf of trained nurses, she says, "We do not intend to be quiet, and shall have a good deal more to say, although it may not be in the columns of The Hospital 'Nursing Mirror.'" It only remains for us to point out that neither we, nor the Birkenhead Guardians, can go behind the certificate which has been given by the authorities of the Chorlton Union Infirmary; but it is open to anyone who has representations to make to communicate with the Local Government Board.

### THE QUEEN'S NURSES AT ROCHDALE.

A gratifying report was presented at the meeting of the General Committee of the Rochdale District Nursing Association the other day. It was stated that the visits paid by the nurses during the last three months were 2,404, the number of cases dealt with being 125. Miss Armstrong, inspector for the North of England under the Queen's Jubilee Nursing Institute, recently visited the home at Rochdale. She stayed for a couple of days, and went with all the nurses on their rounds, thus obtaining an excellent insight into what is going on under their auspices. The result of her inspection was that in a communication to the general superintendent she referred in the most favourable terms to the work. A proposal was made to appoint an additional nurse for the Castletown district, but it will be considered again on a future occasion.

### THE BLUE BOTTLE FLY.

Referring to a note from a nurse in India respecting patients breeding maggots, a Colonial nurse writes: "I was for five years nursing in New Zealand, where bluebottle flies are a source of great annoyance. They abound in millions, and although I have occasionally seen patients admitted with fly-blown wounds I am pleased to say we always managed to avoid such a thing occurring after admission. We were provided by the hospital authorities with a sort of muslin gauze with which we always covered the faces of helpless patients, carefully tucking it round underneath the edges of the pillow. Cases of delirious typhoid, cancer of the jaw, &c., were all protected in the same manner. It gives the patients a rather ghastly appearance, but is infinitely preferable to the state of affairs described by a nurse in India. I have frequently seen fly-blown blankets amongst the 'swags' brought in by patients. Swag is a colonial term applied to the bundle of blankets which the men who tramp the country carry with them for a shakedown. We generally have to store the goods and chattels of these men during their residence in hospital, as they have seldom anywhere else to leave their belongings. I have known shepherds come into hospital for treatment and bring a sheep-dog—of great value to the shepherd—with them. As a rule we get someone to take care of it. People who camp out in the colonies find it necessary to roll their blankets in oilskin or American cloth in the daytime to keep the bluebottles out of them."

### HOW TO HELP A NURSING ASSOCIATION.

The value of a large number of small subscriptions is not always sufficiently well recognised. When a fund is in need of help people are too often apt to jump to the conclusion that the only chance of putting things right is by means of substantial contributions from the wealthy few, and if these are not forthcoming they begin to despair. At a garden fête in connection with the Kendal Home Nursing Association, Mrs. Bagot, in pointing out its necessities, suggested that if a hundred small subscriptions of half-a-crown a year each were given by persons who had never given before, that would make a great difference to the resources; and, by way of a start, she said that her little son would subscribe a couple of half-crowns. This is the right way to help a nursing association. It is not only that very many who could not afford a guinea or half-guinea, can spare half-a-crown, there is an obvious advantage in multiplying, as far as possible, the number of individuals interested in a movement; and we fully expect to learn next year that the result of Mrs. Bagot's appeal to the public of Kendal, who fully acknowledge the excellence of the work done by the nurses, has been highly satisfactory.

### A LITTLE TURK.

A little child, the son of a Turkish officer, was lying ill in the ward of a small English hospital in Palestine. He had spinal curvature and some caries. He was a great favourite with the nurses, and the pet of the patients. He loved to be called "Effendi," a title of respect and superiority somewhat equivalent to "esquire" in English. But some days his ambition rose higher, and he wanted to be called Sardadine Pasha. Sardadine was his Christian name. Every morning, as the night nurse went off duty, she would ask him what he was going to be called that day. Sometimes it would be Effendi; at others, Pasha, Emir, or Moodeer. Once or twice he insisted upon being addressed as Sultan! One day he did not rise to the usual morning question so quickly as was generally his wont. The nurse ran though the category: "Are you Sultan, Emir, Pasha, Moodeer, or Effendi to-day, Sardadine?" To each of which he solemnly shook his head. Then, after a pause, he said, "I am the Vizier." Poor little boy, he imagined that the post of Vizier would suit his capacity—or, rather, incapacity—for he would have nothing to do, except sit at the Sultan's right hand.

### SHORT ITEMS.

Miss Barwell, of the Army Nursing Service Reserve, was one of the witnesses before the South African Hospitals Commission on Monday. She stated that the men never complained.—The sum collected at the annual church parade at Wycombe this year in aid of the Cottage Hospital and Nursing Institution was £51 1s. 6d., a record amount on the occasion.—The matron of Allt-yr-yn Hospital, Newport, Mon., wishes to correct her error in describing the new post of Miss Farrington, who has been appointed night sister, and not night superintendent, at Enfield Isolation Hospital. —Fifty-seven applications have, we understand, been received for the appointment of matron at Ardrossan and Saltcoats New Joint Hospital, Springvale. The choice may not be made for eight or ten days.

### Lectures on Nursing for Probationers.

By E. MacDowel Cosgrave, M.D., &c., Lecturer to the Dublin Metropolitan Technical School for Nurses.

### XII.—DIGESTION.

Milk is called a perfect food, as it contains all the necessary varieties of food, and can support life, as is seen in infancy and in illness. It contains five things—caseine, sugar, fat, salts, water. An ordinary meal contains the same five varieties of food; thus breakfast may consist of egg, bread, butter,

Fig. 21.—Section of Face and Neck.

1, Pharynx; 2, gullet; 3, vertebræ of neck; 4, nasal passages; 5, soft palate; 6, tongue; 7, epiglottis; 8, windpipe.

salts, water. Dinner may consist of lean meat, potatoes, fat of meat, salts, water. In either case the salts are combined in the food, and whatever liquid is taken contains water. The proteids—caseine, egg, lean meat, &c.—are chiefly flesh-formers; the bread, potatoes, butter, and fat are chiefly heat-givers.

The object of digestion is to render food soluble and capable of entering into the system. Food is digested by being cut up small and mixed with various juices, by which it is altered and made soluble; it is then absorbed into the blood. The digestive juices are alternately alkaline (like bread-soda) and acid (like vinegar), and each causes the secretion of the next. Thus saliva is alkaline, and, when swallowed, stimulates the stomach, making it pour out acid gastric juice. The gastric juice afterwards stimulates the intestine, causing it to secrete alkaline juice. The digestive juices contain ferments which carry on chemical changes in the food.

In the mouth food is cut up small, for, if large pieces were swallowed, it would take a long time for the digestive juices to act on them. Here, also, it is mixed with the first of the digestive juices, the saliva; this contains a ferment called ptyalin, which acts on starch, turning it into sugar. This action can be seen in the sweet taste arising in bread that has been for some time masticated.

Swallowing next takes place, and the walls of the stomach get red, and gastric juice begins to flow from the glands. This gastric juice contains hydrochloric acid and pepsin, and acts on the proteids, dissolving them; when dissolved they are called peptones.

The walls of the stomach have two layers of muscular fibres. These, by their contractions, keep the food moving about, so mixing it with the gastric juice and bringing the digested portions in contact with the blood-vessels in the walls of the stomach in order that they may be absorbed. Gradually the proteids get dissolved and the fat gets melted; the starch that the saliva has left undigested remains unchanged. In three or four hours the contents of the stomach become fluid, the pylorus then relaxes, and the chyme (as the partially-digested fluid is called) passes out into the first part of the small intestine—the duodenum. Here it meets with the bile, which is formed by the liver and stored up in the gall-bladder until required, and with the pancreatic juice, which is formed by the pancreas or sweetbread. These flow into the intestine by a common opening, and mix with the contents. The bile is chiefly of use to emulsify the fat —that is, to break it up into millions of tiny globules. Milk is an example of an emulsion; the fat, which is collected into a mass in the making of butter, floats about in tiny particles which reflect light and give milk its white appearance. Once the bile has acted on the contents of the small intestine they become white like milk, owing to the fat having been emulsified, and from its milky appearance it is called chyle. The pancreatic juice acts on all kinds of food, digesting everything that is left. The starch that escaped the saliva, and the proteids that escaped the gastric juice, are both digested by the pancreatic juice. There are glands in the walls of the intestines; these secrete intestinal juice, but this has only very weak digestive power; its chief use is to keep the contents of the intestine fluid.

The walls of the intestine—like those of the stomach—have two layers of muscular fibres, circular and longitudinal; waves of contraction pass down the walls in these layers, and gradually the contents are moved on. As the contents move on two things take place—the remaining portions of the food are digested, and the digested parts are absorbed. Just as in the stomach, the blood-vessels in the walls of the intestine absorb the digested food that comes in contact with them. There are also special absorbent vessels in the walls of the intestines; these are called lymphatics, and through them all the fat is absorbed, as is some of the other food; the lymphatic vessels carry the absorbed matters to the thoracic duct, from which they are poured into the veins. So which ever way food is absorbed it reaches the blood, and is conveyed through the body.

Fig. 22.—The Alimentary Tract.

cs, Fundus of stomach; s, greater curvature of stomach; œ, œsophagus; sp, spleen; py, p, pyloric end and pylorus of stomach; d, duodenum; i, small intestine; l, liver; gb, gall bladder; va, vermiform appendix; c, cæcum; ac, ascending colon; tc, transverse colon; dc, descending colon; ic, sigmoid flexure; r, rectum; a, anus.

### Nursing at Kimberley and on Board the "Britannic."

### A TALK WITH THE SISTER SUPERINTENDENT.

By a Correspondent.

I have just had a talk with Sister Ethel Pope, who has arrived in England for the second time during the war, in charge of sick and wounded on board the transport "Britannic." She was in Kimberley throughout the siege, and had many interesting details to tell about it. Also she had been nursing Boer prisoners, of whom over one hundred and fifty were sent into Kimberley after the fight at Paardeberg.

"They were Cronje's men," said Miss Pope, "and we had among them some notable prisoners—for example, Wessels, and Steyn (a relative of the president), and Ferriere, a field cornet, I think. When Lord Methuen sent them in he said he wished them to be treated just as if they were our own men, in the cause of humanity, and we gave them every possible care and attention."

"What kind of patients were they? Difficult to manage?"

"No, very nice indeed. They were all cases for surgical treatment except five, who had enteric."

### Hairbreadth Escapes.

"The siege was a most terrible experience," continued Miss Pope. "If I had known what was before me when I went out, I don't think I could have faced it."

"Did you go out intending to do army nursing?"

"Oh, no. I went by the advice of a cousin who is a doctor in South Africa. And then I joined Sister Henrietta's Private Nursing Home in Kimberley; her staff is composed of English trained nurses, whom she sends out to private cases. She was for about seventeen years lady superintendent of the Kimberley Hospital."

"And you were there when the siege took place?"

"Yes; my room was on the outside wall, facing the point from which the shells were coming, and it was so terrible that Sister Henrietta insisted on my moving to a room in the middle of the building. One of the nurses, who was just going off duty, had a fearful experience; a shell dropped just in front of her, but it did not burst. They have kept it as a relic of the siege."

"There must have been many narrow escapes?"

"Yes, I could tell endless stories about it. One lady, who had gone to lie down in the afternoon, was horrified by a shell which passed through the bed on which she was lying; it went through the floor without bursting."

"The saddest death," Miss Pope thought, "was that of the chief engineer of the De Beers Mines, who was killed by a bursting shell. The fact that the funeral procession was shelled all the way to the cemetery and all the way back to the town proved the presence of traitors in Kimberley, for all funerals took place at night. They did not fire while the service was proceeding."

### Nursing in a Skating Rink.

"When the siege was raised Sister Henrietta's nurses were put in charge of various public buildings which were commandeered for the sick. I had charge of the skating rink. Every possible place was used, churches, chapels, schools, &c."

"A skating rink would certainly make a novel hospital ward. How did you arrange about beds?" I inquired.

"We had mattresses for the worst cases, and some just had stretchers. They only stayed with us three weeks, and were then sent to Cape Town by ambulance train."

"What are the trains like?"

"They are most comfortable, and beautifully fitted up. The beds are like the bunks on board ship. A doctor and two Nursing Sisters are in charge."

### About the "Britannic."

"And now," I said, "will you tell me something about your voyage home? What kind of ship is the 'Britannic?'"

"She belongs to the White Star Line, and runs between Liverpool and New York. She was chartered by the War Office; and we were fortunate in having her own doctor, Dr. Tribe. I cannot say too much in praise of the entire ship's company—captain, stewards, and everyone were so kind and attentive, and did all that was in their power for our comfort."

"What was the extent of your nursing staff?"

"I had three nurses; one, Miss Hardman, has had a very great deal of experience, and though she is not certificated, she is invaluable, and a splendid nurse in every way. I have the utmost possible regard for her. She has done a great deal of district nursing, and is very capable indeed. The nurse who had charge, under Dr. Tribe's assistant, of the surgical cases, had been nursing since the war began. The third was a probationer, quite fresh to the work, but a born nurse, and such a bright, companionable girl. This is her first visit to England, and she is enjoying everything thoroughly; even a ride on the top of a bus is the greatest delight to her."

"Would you like to say anything about the orderlies?"

"They were civilians, and untrained. A corporal of the R.A.M.C. helped in taking down the prescriptions when I went the rounds with the doctor, and the stewards acted as orderlies. They were the greatest possible help."

"How many men were there on board?"

"From five to six hundred Tommies and sixty officers."

"But these were not all ill enough to require nursing?"

"Oh, no; but all were invalided home. Seventy-one went to Netley, and the others were sent to the discharge depôt. The number requiring hospital treatment was from 20 to 25. There were about 20 beds for medical cases, and 50 for the worst convalescent cases; these were under the care of the corporal and orderlies, but if they got very bad they were sent to me for my immediate care. We had relapses in enteric and dysentery cases, and we lost one man. He died, with a temperature of 106, of pneumonia, having come to us suffering from asthma. I never left him at the last for more than five minutes, but I am sorry to say all we did for him was unavailing. I would have gone to the funeral to show my respect, but I couldn't, it had upset me so. It was at eight o'clock in the morning when they buried him, and I think they wanted it to be over before I was up—they all knew how I felt losing him."

"How did you arrange the wards?"

"The large saloon, which was intended for the largest ward, was so far from the dispensary and gave us such a long distance to walk that we used the separate cabins for the worst cases; and if a man was very ill we removed the bunk over him. Of course it made a lot of work going to and fro, but it was more convenient, and the ship is not intended for a hospital, so we could not have it all as convenient as we should have liked."

"And were the arrangements about the food good?"

"Excellent. Everything was splendidly managed, and we had only to say what we wanted and it was supplied at once. Of course no food was supplied to the hospital except under my supervision; I attended to all the diet myself under Dr. Tribe's direction. The nursing staff dined with the officers at the doctor's table."

"Are you going back again?"

"Yes, I go back in the "Nubia" about the 6th. And then I should like to go to China. I was at Daly's Theatre last night seeing 'San Toy,' and one of the songs so touched me that I felt I must go back at once; I have a feeling that I couldn't stay away as long as there is work to be done."

### Nursing in Sanitoria for Consumption.

By Jane H. Walker, M.D.Brux., Physician to the New Hospital for Women, and Medical Superintendent of the East Anglian Sanatorium.

### III.—THE TRAINING FOR NURSES.

Sanatorium nursing, from a nurse's point of view, possesses the great advantage of not being specially arduous, and therefore it can be undertaken by women who are too delicate to go through the regular hospital course. This brings me to the subject of training. Far be it from me to say, as one doctor at a sanatorium has said, "I want automata, and not nurses," and who therefore employs people of the regular servant class; but at the same time the ordinary hospital-trained nurse is not of much use in a sanatorium for better class patients. She is too apt to be full of preconceived ideas, and if she has been at a chest hospital is imbued with the notion that consumption is incurable, and does not realise that a large part of the cure depends on cheerful surroundings. But some training is necessary. It makes a good deal of difference to a patient to have his bed made properly, to be skilfully washed in bed by an experienced hand, and to be otherwise attended to by someone to whom the whole thing is a matter of course. All these things, which are factors common to all nursing, can, I think, be learnt in six months' training in an ordinary hospital, and such is the plan I have adopted in the nursing department of my sanatorium. A very useful thing which is being gradually evolved at the present time is a training school for nursing on the rational method of treating consumption. Not infrequently, for one reason or another, it is necessary for a consumptive patient to remain at home, and for such it is advisable to provide a so-called "open-air" nurse.

Of course, in choosing nurses for consumptives one should remember that they should be of specially cheerful dispositions, should be possessed of considerable tact, and should recognise the fact that consumption is not an incurable disease. By some people stress is laid upon the importance of the nurses being physically strong. On the other hand, it is sometimes considered an advantage for the doctor at a sanatorium to have been gone through the necessary training and discipline by having been a patient himself. There is, perhaps, something to be said for this idea, and, whatever it is worth, it applies at least as much to nurses as to doctors. In staffing a paying sanatorium with nurses one nurse to every six or seven patients should be allowed for, and if many patients are entirely in bed occasional extra help will be probably required.

### Nursing in Sanatoria for the Poor.

At the risk of repetition, attention must again be directed to the fact that consumption is not an ordinary acute illness, short in its duration; it is a long and tedious illness, requiring at least six months for due treatment. It can easily be imagined that to take working men or women away from work, patients whose interests are, to say the least of it, very limited, who are used to waiting on themselves, to living on scanty diet, or at any rate on plain food, and to place such patients in a luxurious home replete with every comfort, where they live like fighting cocks, and are waited on hand and foot, and where they must stay for a considerable time, is demoralising in the extreme, and renders them practically unfit to follow their former occupations when they are well enough to return to them. This being so, the danger must be avoided by arranging Sanatoria for Poor Consumptives on the simplest possible lines. The sanatorium must be furnished as sparsely as can be, the food must be as plain as is consistent with the due proportion of constituents required for a dietary for consumptives, and whenever the patients are able they must give what assistance is required in the house and garden. All these points make the arrangements in a Sanatorium for the Consumptive Poor very different from those found to work best in a well-paying establishment. Fewer nurses will be required, and perhaps these should be of rather a different kind. Thus, in a sanatorium of say eleven beds a nurse who is working matron and housekeeper, with a young probationer under her and a servant to come in by the day, is all the staff required. (In a well-to-do place the total staff of all kinds is about one to every two patients.) All but the absolute scrubbing of the house can be done by the patients. But great tact and patience is necessary to deal satisfactorily with this part of the work. We all know that it is far easier to do things ourselves than to make others do them, and far more is this the case when the persons we wish to make work are, firstly, not feeling very robust, and, secondly, are resenting our efforts with all their powers. They are specially apt to do this where they pay, and more still where someone else is paying for them. And yet it ought to be done if the last state of the patients is not to be worse than the first.

Of the household work, dusting with a wet duster, washing up crockery, cleaning boots and knives, laying the table for meals and clearing it again, and most of the necessary mending and sewing, can be done by the men and women patients, and such help as they can give should be required of them. Equally, whatever garden work can be done by men patients should be enforced. It must be remembered that, provoking as well-to-do consumptives are in grumbling over their meals, they are not to be compared with poor patients. This must be faced and reckoned with by any nurse working in a sanatorium for the consumptive poor. The food should be as far as possible of such a kind that it is sufficiently within the means of the patient to procure for himself when he leaves. Suet pudding, haricot beans, lentils, bacon, pease pudding, dripping, cheese, and so forth, are articles of very high dietetic value, and can be obtained at comparatively cheap rates. A great deal of tact will be needed to make the average person of the artisan class think he is getting his money's worth with such a diet. His one idea of food, as far as I am able to judge, seems to consist of anything cooked in a frying pan, such as fried rump steak. But that idiosyncrasy must be prepared for. Statistics as to results obtained in sanatoria for poor consumptives are highly encouraging, being little if at all inferior to those got in better class sanitoria. Moreover, if the money for the building and furnishing is found the institution—20 to 30 patients paying 12s. to 15s. a week—can be made, not only just to pay expenses, but to pay a dividend of 2-2½ per cent.

### Nursing in Foreign Sanatoria.

This part of my subject is rather like describing the snakes in Iceland. Nurses, as we understand the term, do not exist. The attendants on the patients are servants only, and they have no training or nursing experience whatever. This at least is so at all the sanatoria with which I am acquainted, either by personal observation or by repute. They are very good and kind, and do their ignorant best, and very timid and anxious they are, poor things, when they come into contact with the graver and more serious aspects of the disease. When the patient is up and well they clean his room and make his bed; when he is ill they, in addition, bring his food, and wait on him more or less, making his bed or not as he wishes. When he is too ill to have any visitors, one of them—with the exception, of course, of the doctor—is his only comfort. The servant often does not know his language, and he often does not know more of hers than suffices to ask for the barest necessaries.

I cannot help feeling that one pull English sanatoria—not imitating slavishly everything German—have over foreign sanatoria, will be found in the much greater comfort existing in the nursing department.

### The Nursing of Paupers.

INTERESTING REPORT BY A GOVERNMENT INSPECTOR.

In the twenty-eighth annual report of the Local Government Board, 1898-99, the question of workhouse nursing is touched upon by several of the inspectors. But Mr. Baldwin Fleming, the inspector for the district comprising the Union Counties of Dorset and Southampton and part of Wilts and Surrey, deals with it at length, and sets himself resolutely to remove the ignorance which prevails upon the subject. We give the following extracts from his report:—

The Nursing Order.

"On the whole," Mr. Fleming says, "probably as much progress has been made as could reasonably have been expected towards carrying out the Nursing Order. Much more might have been done had there been a better supply of competent nurses willing to take workhouse situations, or, in other words, had guardians been willing to pay them and treat them better. The subject is a difficult one and can only be dealt with by degrees. It is quite certain that young hospital nurses, who are properly ambitious to rise in their profession, cannot be expected to take situations in small country workhouses, and it is much better that they should not do so. This does not at all mean that inferior nursing should be accepted in union work. Any proposal to admit nurses, as nurses, for workhouse places with inferior qualifications should not be entertained for a moment, and any idea for admitting second class nursing in the smaller houses should equally be repudiated. The nursing given to workhouse patients should be the very best nursing that they can have. A great many of them, however, do not need attendance requiring the physical qualification necessary for nurses who have charge of critical acute cases, but it is a mistake to suppose that workhouse situations give less scope for good and useful work than other branches of the profession do. The kindly care of senile decay is as meritorious and as grateful a duty as is the relief of any other form of sickness or distress."

Average Cases.

"Here are the cases on the workhouse medical relief book of an average country workhouse: 14 senile decay, 4 debility, 1 paralysis, 1 rickets, 1 chronic ulcer, 1 chronic bronchitis, 2 ulcerated leg, 1 rheumatism, 1 rupture, 1 blind."

"Here are the cases in a larger rural union: 37 males, of which 6 were paralysis, 3 debility, 2 senectus, 4 epilepsy, 3 cough, &c., 3 unsound mind, 3 bronchitis, 1 hip disease, 1 goitre, 1 ulcer of leg, 1 incontinence, 1 gangrene foot, 1 cancer, 2 fits, 1 effects of heat, 1 diarrhœa, 1 syphilis, 1 asthma, 1 eczema; 37 females, of which 10 were senectus, 5 ulcer of leg, 5 paralysis, 2 rheumatism, 1 chronic bronchitis, 1 epilepsy, 1 Pott's disease, 1 contusions, 1 cough, 1 hœmorrhoids, 1 debility, 1 procidentia, 1 idiot, 1 diarrhœa, 1 albuminuria, 1 enciente, 1 neglect, 1 crippled, 1 weak spine."

"It is clear that many of the above cases require a great deal of care and nursing, but that a large proportion of them are not cases of an interesting description which ambitious young nurses would care much to have to deal with, nor which would further their professional advancement. In the smaller unions they are just the cases which could be well attended to by a trained nurse past the age of her fullest activity, if she had comfortable surroundings and competent paid and responsible assistants under her. Of course, an acute case may be brought to a small country workhouse at any time, but it is much better to nurse such cases with thoroughly trained younger nurses, obtained for the purpose from institutions for the supply of skilled nursing, who leave as soon as the necessity for their services ceases. If there be rooms always ready, and guardians have an agreement with a nursing institution to send nurses immediately upon requisition, any demand beyond the power of the normal staff can readily be met."

Portsea Island Union Infirmary.

"Nurses' objections to workhouse situations ought very shortly to be overcome in such establishments as the Portsea Island Union Infirmary. The provision made for them is excellent, and the guardians and officers wish the conditions of the nursing service to be made as pleasant as possible. The number of cases is large and of great variety, including many surgical patients. The detached imbeciles' blocks are included in the sick department, which affords opportunities of training in mental nursing and the care of dements such as are not often available in general hospital practice. Probationers are trained under a thorough course of instruction. Nurses and probationers are encouraged in every way, and well looked after; they have regular and sufficient leave, and successful efforts are made to render life cheerful and good for them. There is no reason why the position of the nurses here, both as regards professional possibilities and the amenities of life, should be regarded as inferior to that of nurses in general hospitals of good repute. When the Southampton Guardians complete their infirmary arrangements, the training and position of the nurses will probably be organised upon similar lines."

The Average Country Union.

"Between these important institutions and the smallest of the rural workhouses there is a very varied series of requirements, and it is with regard to the average country union that difficulty is experienced—and upon these grounds. Usually, where the number of patients is small there is only one nurse. The guardians are apt to grudge an adequate salary. The class of case is uninteresting from the ordinary professional point of view. The nurse seldom sees any doctor except the workhouse medical officer. The arrangements for leave and recreation are imperfect. A nurse ought to have from one to two hours' regular leave every day, a long half-day once a week, every alternate Sunday, and three weeks (at least), or a month's consecutive holiday during the year. She ought not to be required to perform menial duties, and she ought not to be made to depend upon pauper assistance (which is always unreliable) for their performance. That is, she should have a paid wardswoman upon whom she can rely. Unless a nurse can rely upon having her orders carried out with regard to subordinate matters she has to look into them herself, and assistance is of little real saving to her."

The Economies of Guardians.

"Now, in country workhouses these important matters are frequently disregarded, and, even worse, there is a great tendency on the part of guardians who have never been ill to think that nurses have very little to do, consequently attempts have been made to underpay them, and to place all sorts of duties outside their own work upon their shoulders. Thus, in one union a nurse was required to bathe the female vagrants; in another, to attend to and dress in female admissions to the workhouse; in another, the assistant nurse was called upon to help the matron in her stores. A further point is that the dietaries of the nurses, and, indeed, of the other workhouse officers, are frequently extremely monotonous. The same articles are supplied week after week, and no sufficient allowance is made to ensure the variety, without which the best of food palls. This matter is entirely within the Guardians' discretion. They can arrange the officers' dietary to their liking without reference to the Local Government Board. It is astonishing how reluctant some Guardians are to give pleasant feeding to their officers. In one union they cut down the officers' bacon from 7d. a lb. cured to 4d. a lb. 'green,' because some of the Guardians did not see why the workhouse officers should have better bacon than they had—failing to measure the difference between the healthy appetite of the out-of-door worker and the often tired palate of the nurse who has been for hours at work in the sick room. An excellent nurse left in consequence of this little economy. It was the "last straw." Again, the cooking and serving of the nurses' meals is often objectionable, and no comfortable arrangements are made for service upon her—in fact, she often has to wait upon herself, to do her own room, and is fortunate if she have not to do much of the menial work in the wards also; such assistance as she has is from pauper helps, who are constantly changing, and frequently unpleasant and incompetent. The ward work is often made additionally and unnecessarily trying by the absence of proper facilities for saving labour—such as hospital sinks, baths fitted with hot and cold water, separate stores for linen, bedding, crockery, &c., under the nurses' own control, the lack of adequate classification wards or separation rooms, and various other disadvantages which still exist in many of the less well-found rural houses, notwithstanding the great improvements made of recent years."

HOW TO EFFECT IMPROVEMENTS.

"A great many of the objectionable features are unnecessary, and are being removed. Where there are lady guardians, and, happily, now there are many, they can be of the greatest service to the nurse in promoting her comfort and putting her in the way of making her recreation time pleasant. The fact that in the small country houses the nurse is under the matron ought not, in itself, to be an objection. Nurses who take situations of this kind have not always been in independent positions beforehand, so that it is not new to them to be under a superior officer. The comfort of a nurse must depend very much upon her treatment by the master and matron. Those officers, generally speaking, are kindly folk, who have no object in treating a nurse badly, but, on the contrary, would naturally be disposed to treat her well if she be willing to reciprocate their good disposition. They are, however, handicapped upon many of the above points by the limited means and staff at their disposal. The necessities of an average rural workhouse would seem to require dealing with somewhat in the following manner. The Guardians should have a nurse of middle age who has, at a former period, been thoroughly trained, but who has finished with hospital work and the more active branches of her profession. Under her there should be a competent paid wardswoman, who can be relied upon by the nurse to carry out her instructions; and, where the number of patients is at all considerable, there should also be a paid servant under the control of the nurse, who should perform the more menial duties in the sick wards, and might wait upon the nurse also. This would generally be sufficient for normal conditions, but would not provide for day or night nursing upon acute cases requiring constant attention. This need should be met by having rooms always ready for additional trained nurses, to be obtained from a nursing institution. By means of a little kindly thought and consideration a great deal may be done to make a nurse's life a pleasant one, even in the most unlikely workhouse. There are generally lady visitors, there are often lady guardians, there are always guardians' wives and daughters. If these will give the nurse their sympathy and companionship, they can help the master and matron to relieve the loneliness and brighten the dull hours which render a country nurse's position trying and undesirable. The usual objections will be raised that additional officers will have to be appointed and additional cost incurred in order to carry out the above suggestions. This is so, but the Board's nursing order cannot be obeyed unless such officers are appointed as will prevent the use of pauper help in nursing, and the money spent upon what is required for the proper care and treatment of the sick is money which is well spent, which must be spent, and which is a thoroughly legitimate and praiseworthy expenditure."

IMPOSSIBLE CONDITIONS.

"Where the above particulars are disregarded, and the nurse is always on duty, where her food is unsavory, no service is provided for her, no assistance is given in her wards, where the necessary articles for use are not freely supplied and under her own control, where the absence of help during the night undoes her good work during the day, where her recreation and amusement are unconsidered, and where she has no congenial companionship, and nothing but hard and thankless toil from morning till night, from week's end to week's end, a good respectable nurse cannot be expected to stop, ought not to stop, and happily will not stop. It is a matter of complete satisfaction to me that guardians who grudge pay and comforts to nurses find it difficult to get them. The difficulty will raise the character and status of workhouse nursing, and perhaps it is not too much to hope will lead to such improvement in the conditions of the service as will make workhouse situations desired rather than shunned."

THE EXTRAVAGANCE OF INEFFICIENCY.

"Good surroundings for the nurse are important for the nurse's own comfort, but infinitely more so for the comfort of her patients. An ill-fed and ill-found nurse will not be good tempered and pleasantly disposed, which it is most necessary that a nurse should be. The nurse suffers, but the patient suffers more. The Board's Nursing Order requires that the sick poor shall be nursed (unskilled attendance is not nursing), and therefore guardians must bring their nursing arrangements up to such a standard as will ensure proper pay, proper comfort, proper surroundings, proper assistance, and a due share of the pleasures of life for their nurses. When this is done workhouse situations will not be inferior to other places, and will need neither peculiar qualifications nor special virtue on the part of those who take them. The solution of the difficulty of obtaining good nurses for country workhouses is in the guardians' own hands. If they will pay them adequately and make the conditions of their work good for their patients and pleasant for themselves (the two things are really synonymous), there is not likely to be any lack of fitting candidates. If the guardians will look rightly at the subject, they should see that the duty, to save suffering to the sick, is a higher duty than to save money for the ratepayers, quite apart from the fact, pithily put by Miss Florence Nightingale, that 'there is nothing in the long run more extravagant than inefficiency.'"

### Presentations.

CHORLTON UNION WORKHOUSE.—On Friday evening last a social gathering of the officers of the Chorlton Union Workhouse took place. The object was to make a presentation to Miss M. Beard before her departure to take up her duties as superintendent nurse of the Birkenhead Workhouse Infirmary. The Master (Mr. J. Firth), whilst expressing his regret at losing such a valuable officer, wished Miss Beard every success in her new appointment. He then called upon the Matron (Mrs. Firth) to make the presentation, which consisted of a gold curb bracelet from the officers of the workhouse, a portmanteau from the nurses of her own department, and a silver serviette ring from her successor, who was formerly one of her nurses. In the course of her speech the Matron, who is herself a hospital-trained nurse, said she thought the Birkenhead Guardians were to be congratulated on their choice of a superintendent nurse, as she felt sure that Miss Beard would always endeavour to do her duty well, and never bring discredit on the certificate she has obtained from the Chorlton Guardians. In asking Miss Beard's acceptance of the bracelet, she hoped that the links in the chain would remind her in times to come of the many friends and well wishers she is leaving behind her at Withington. The Rev. T. Horne, chaplain, in returning thanks on Miss Beard's behalf, said he would like to testify to the admirable way in which she had always discharged her duties during her six years of office. Miss Beard was also the recipient of a beautifully bound Prayer Book from the Rev. Father Burke.

RUCHILL HOSPITAL, GLASGOW.—On her departure from the Middle Ward Hospital, Motherwell, to Ruchill Hospital, Glasgow, Miss Adams, matron, received many indications from all sides of the esteem in which she is held. The recognition of the Middle Ward Committee was transmitted by the clerk in the following terms: "I am also instructed to express the committee's regret at losing your services, and to convey to you their high appreciation of these services while you have been in their employment." A handsome dressing-case was the present from the staff of the hospital as a token of their esteem, while Dr. Wilson, Medical Officer of Health for the county of Lanarkshire, and Mrs. Wilson gave as a mark of their regard a valuable silver cake basket, fitted with morocco case. All these are excellent memorials to the admirable work accomplished by Miss Adams in her late sphere.

BANGOR AND DISTRICT NURSING SOCIETY.—Miss Kathleen Martin, who for three years has acted as nurse under the Bangor and District Nursing Society, and has just been appointed to a post in Dublin, has been presented with a purse of sovereigns and an illuminated address from her numerous friends and well-wishers in and around the town. The Dean of Down was one of the speakers on the occasion, and he, and others, paid a warm tribute to the assiduity, the patience, and the skill shown by Miss Martin during the three years she had been at work among the poor.

### On the Care of Patients Before and After Operations.

Abstract of a Lecture to Nurses given at the City Orthopædic Hospital on June 26th, 1900, by J. Jackson Clarke, M.B.Lond., F.R.C.S.

Diet and Management of Bowels.—The diet and management of the bowels before operations are points of importance. If a patient is given an anæsthetic and has a full stomach vomiting will probably occur and various evils may result, *e.g.*, the patient may inhale some of the vomited matters into the wind-pipe with even fatal consequences, and the least inconvenience that arises from this undesirable accident is an interruption of the operation, and in some cases a soiling of the wound. If the patient is allowed to go into the theatre with a loaded rectum other unpleasant consequences may occur. When an operation is planned to be done soon after breakfast-time I like the patient to have a dose of castor oil of from 30 drops to two tablespoonsful according to the age and strength of the patient; infants under one year require something less than a teaspoonful, and patients over that age something more.

The time at which the aperient should be given varies according to the case. If the operation is to be done in the forenoon the aperient is to be given after breakfast in the preceding day. If the operation is to be done in the afternoon the aperient may be given at bed-time. In those cases in which from one cause or another this preparation cannot have been carried out, an enema must be given at least one hour before the patient is operated on. In infants six to eight ounces of soap and water with a little olive oil; in adults, one to two pints of soap and water, or if the time is shorter three drachms of glycerine should be administered.

With regard to feeding. The aim is to have the stomach empty without the patient being too much weakened. After a meal of moderate dimensions the stomach in adults empties itself in five or six hours, therefore if the operation is to be done after midday the patient may have a breakfast at eight a.m. The best time for operating is, no doubt, rather early in the morning, and when an operation is done, say, at nine a.m. the patient need have nothing after supper in the previous evening. If the operation is not to be done till later a cup of broth or beef-tea, or of weak freshly-made tea with a dash of milk in it, may be given four clear hours before the operation. Milk should be avoided, since it digests slowly.

Nutrient Enemata.—In patients exhausted by disease, and in cases where the operation is to be severe, it is advisable to prepare for a course of rectal feeding to begin immediately after operation. In such cases a wash-out enema of soap and water (made by rubbing up Castile soap in hot water until a thick lather is formed) should be given three hours or more before the operation is to be performed. Some good beef-tea and some well-peptonised milk are also to be got ready beforehand. For an adult each enema should consist of one ounce each of beef-tea and peptonised milk, the yolk of one egg well beaten up, and a dessert-spoonful of brandy. For a child of twelve half this amount is enough. Each enema should be thrown as high as possible into the rectum. This is easily effected by fixing about four inches of a rubber catheter to the end of the enema syringe. When the operation is to be a severe one a nutrient enema may be given half an hour previous to operation. These enemata should be given with a minimum of disturbance to the patient, and they should be administered every two, three, or four hours, according to the requirements of the case and the tolerance of the patient.

Sickness after an operation is due as much to the effect of chloroform or ether on the brain as to any condition of the stomach. It must be distinguished from sickness due to irritation of the stomach by foul matters in cases where there has been fæcal vomiting; it must also be distinguished from cerebral vomiting, from meningitis, or cerebral tumour. The patient should rinse the mouth out frequently with warm water; in some cases sips of hot water, in others ice-pills give relief. Sometimes a patient is greatly relieved by sips from a tumbler of hot water containing 15 to 20 grains of sodii bicarb.

Shock.—Shock after operations is of reflex character. You are all familiar with the condition of a person severely burnt, or severe physical injury of any kind. The patient is pale because the heart is acting feebly, the pulse also is rapid and weak, the skin is cold and clammy, he may be conscious and answer questions, but he is apathetic, and in severe degrees of shock pain is not felt. To prevent shock the patient must be kept as cheerful as possible in order that mental shock—fear of operation—is not added to the necessary shock of the operation. Cold increases shock, hence the need of protecting the patient as far as possible from unnecessary exposure during the operation, and making him warm in bed afterwards. When there is severe shock, the foot of the bed should be raised on blocks; very warm nutrient enemas should be given, and the amount of brandy in them may be increased to one ounce. The bed should be warmed by hot bottles, and these should be wrapped in flannels and placed near, not touching, the patient. If great care is not exercised in this, extensive blisters may form and add to the shock and prolong the convalescence of the patient. If the shock is prolonged the surgeon should be informed, so that he may judge of the necessity for other measures.

Food after Operations.—No food should be given by the mouth for four hours or longer after ether or chloroform-anæsthesia. A cup of tea is often best relished. Broth or beef-tea may be given, but not milk, as the first meal, for the reason given above.

Hæmorrhage.—Loss of blood may occur from arteries, from which it escapes in jets of bright blood; from veins, whence it comes as a steady flow of dark blood; or from capillaries, as an oozing from the surface of a wound. The amount of blood that escapes from a wound varies greatly. A person may have a limb torn off without there being any hæmorrhage, though large veins and arteries are divided. This is because when blood-vessels are torn or twisted apart the more elastic and brittle inner lining curls up, whilst the more fibrous outer coat is drawn out like a night-cap over them. After a large amputation there is necessarily a certain amount of capillary bleeding, and as soon as the dressing is soaked by blood at any part notice should be given to the surgeon. At the end of an operation the wound may be quite "dry," as it is termed, but as soon as the patient gets warm in bed an artery may begin to bleed owing to the heart recovering its force. In this event in case of a wound the dressing will not only be soaked quickly, but blood will drip from it. This arterial hæmorrhage requires immediate attention. Pressure on the main artery of the limb should at once be made, the limb elevated, and the house-surgeon summoned with all urgency. After some operations, *e.g.*, abdominal section, certain operations on the rectum, &c., the bleeding may occur internally, without any blood appearing on the dressing or elsewhere. In such cases blanching of the lips, rapid weak pulse, and deep and rapid respiration (air-hunger) are indications of danger;—the last symptom serves to distinguish hæmorrhage from shock.

### Important to all Trained Nurses.

THE NURSES' WHO'S WHO.

DURING the next two or three weeks the forms to be filled up and corrected for the 1901 edition of "Burdett's Official Nursing Directory" will be sent out, and we would again most strongly urge that these should be carefully attended to and returned, without delay, to the Acting Editor, "Burdett's Official Nursing Directory," 28 and 29, Southampton Street, Strand, W.C.

In our issue of January 13th last we pointed out at some length how important to nurses it was that they should take immediate steps to have their names entered in the "Directory," and the necessity was brought home to us with all the greater force at the time in consequence of the many inquiries we received relating to the nurses going to South Africa. We were asked—and the question was a natural one—why an apparently invidious distinction was made, by giving the full particulars of the career of some of the nurses, while in other cases only the names were mentioned? The reason was that the names of those nurses concerning whom no particulars were given were not in the "Directory," and consequently nothing was known about them.

A little consideration will show how exceedingly detrimental it is to a nurse when reliable information as to her training and career is not easily available. Sickness in a house seldom gives much warning of its coming. A nurse's services are required suddenly and immediately, and from the names of those who are engaged in private nursing which appear in the "Directory," it is easy to make a selection. The nurse whose name is not entered there suffers accordingly. Or, for a further example, take the case of a doctor who has had a nurse recommended to him. He will naturally want to know something of her training and career, and if, on consulting the "Directory," he finds that her name is not there, it is more likely than not that he will pass her over and select one whose name is. In short, the position is this: those nurses who through carelessness omit to have their names entered in the "Directory," which has obtained to the rank of a recognised authority, run the risk of sharing the fate of those unqualified individuals whose names would not be admitted to its pages on any consideration whatever.

So far, then, as those nurses whose names are not entered are concerned, it is obvious they should at once take the trouble to rectify the omission. And here it may be as well to point out that no trouble or care on the part of the editor is sufficient to insure the "Directory" being a complete record without the co-operation of the nurses themselves. A form of application is sent to every nurse whose name is obtainable, and the work of getting these names together from every available source is one of great magnitude; but it stands to reason that the list so obtained cannot be exhaustive, and the nurses who do not receive forms of application should write to the Acting Editor for one, or fill in the specimen one which we append. We repeat that the forms should be attended to and returned at once, for to delay means very often to forget altogether. As it was pointed out in the preface to the last edition, no sooner had the article, to which we have referred, appeared, than a large number of applications was received, many from the very nurses who had already had forms sent them which they had omitted to return.

With regard to the nurses whose names are already in the "Directory," and who will in due course receive a proof for correction, we would emphasise the fact that a great deal of trouble would be obviated and time saved if nurses would make it a rule to notify any change of address or additional particulars of their career to the Acting Editor. Many of the letters sent out are returned through the post, marked "Not Known," the nurse having changed her address during the year, and it is often impossible to obtain the new address; the consequence is that the name appears in the new edition with an asterisk against it. A word concerning the asterisk which is put before some names is perhaps advisable, for it is possible that some nurses on receiving the proof and seeing that the details given are correct, may think it a matter of small consequence whether the proof is returned or not. It is of great importance, for an asterisk is placed against the name of every nurse from whom no return is received, and means that there is no guarantee that the particulars given are correct beyond a certain date. How necessary this precaution is will be readily understood when it is remembered that the "Directory" is a compilation in which every statement made with respect to a nurse has either been verified by competent authorities or derived from official sources. The position which the work holds as an absolutely correct authority clearly could not be maintained if it were taken for granted that the address and particulars given of a nurse were correct simply because nothing had been heard to the contrary.

It may be truly said that nothing is left undone, no trouble is spared, to make "Burdett's Official Nursing Directory" each year a more complete record, and therefore more widely useful, not only to the nurses themselves, but also to the medical profession and to the public generally. With this object in view, the Acting Editor is always ready to give any assistance in his power, and to answer any question or advise in any difficulty to the best of his ability.

The following is the form of application already mentioned:—

To be filled up by the Nurse to whom the particulars relate.

(1) Name in full, and Address—
(2) Present Occupation— Date of entering upon it—
(3) Particulars of Nursing Career in the following form:—
  Probationer, at Hospital from , to
  Staff Nurse, at Hospital from , to
  at from , to
  at from , to
  at from , to
(4) Certificates held (stating in each case at what Hospital or Institution training was received, and giving the dates of the commencement and end of training):—
  General Training Certificate received at Hospital for years' training.
  Midwifery Certificate—
  L.O.S. Certificate—
  Monthly Nursing Certificate—
  Massage Certificate—
  Medico-Psychological Certificate—
(5) List of Medals and Badges held, if any—
(6) Any qualifications or experience beyond what is given above?

### To Nurses.

WE invite contributions from any of our readers, and shall be glad to pay for "Notes on News from the Nursing World," or for articles describing nursing experiences, or dealing with any nursing question from an original point of view. The minimum payment for contributions is 5s., but we welcome interesting contributions of a column, or a page, in length. It may be added that notices of entertainments, presentations, and deaths are not paid for, but, of course, we are always glad to receive them. All rejected manuscripts are returned in due course, and all payments for manuscripts used are made as early as possible at the beginning of each quarter.

### Everybody's Opinion.

[Correspondence on all subjects is invited, but we cannot in any way be responsible for the opinions expressed by our correspondents. No communication can be entertained if the name and address of the correspondent is not given, as a guarantee of good faith but not necessarily for publication, or unless one side of the paper only is written on.]

### PROBATIONERS AND THE THEATRE.

"Alpha" writes: I observe that the matron of Kent and Canterbury Hospital, in an interview reported in your last issue, states that she very much objects to the probationers going to the theatre at night. She, however, to some extent, qualifies her opinion by the use of the word "late," for there is undoubtedly a difference between the leading London theatres and some of the provincial houses. I do not know what sort of performances they have at Canterbury, but in certain of the smaller towns the tone of the theatre still leaves a great deal to be desired. At the same time, it would be rather hard on probationers if it were a rule to object to them going to the theatre, while the other nurses went without demur. And then, as to the question of age. The matron of Canterbury Hospital says that she was only alluding to "the young nurses." But probationers are by no means always very young girls, and it does not at all follow that the nurse who is fully trained is a more discreet woman than the one who is being trained.

### SHOULD NURSES WEAR BONNETS AND CLOAKS IN HOT WEATHER?

"Pavo" writes: Why should nurses wear them? Is it their own choice, or does the matron of the institution to which the nurses belong insist on bonnets and cloaks being worn? or is it a Paganistic idea, so to speak, that nurses wear them now because nurses in the past have worn them? We do not see ordinary people wear a lot of superfluous clothing in hot weather, yet the people who really work hard, in ward, theatre, and district, one constantly sees in dark blue cloak and bonnet (which, next to black, is the hottest colour to wear). I think it should be optional. Many people will say that nothing beats the bonnet and cloak. I think, personally, nothing is neater than well fitting uniform dress, collar and cuffs, and plain sailor hat; apron not to be worn except when attending a case. Many nurses in hospitals do not, I am persuaded, go out of doors as often as they ought because of changing their clothes after leaving the wards. The time passes so quickly when one has to change for ordinary clothes and change again for the wards, and so they miss the relaxation of leaving their surroundings for a time. A sailor hat is quickly donned, and if objections are raised to it on the ground that it does not look professional I may plead that all reforms must have a beginning.

### THE OPEN-AIR SYSTEM IN A PRIVATE HOUSE.

"Nurse M." writes: Having read an interesting letter in a recent number of the "Nursing Mirror," by "Nurse M. O." on the above subject, I should be glad to tell her some of the experience gained by a patient of mine. I had nursed her through an illness of congestion of one lung, accompanied by hæmorrhage, and as soon as possible she was taken to an open-air sanatorium, where she improved in a most remarkable manner in a few weeks. Being unable to stay there longer, she is following the treatment in her own home, and her doctor declares her lung to be in a favourable condition. I think that "M. O." makes one mistake in her routine, namely, in joining the family at the mid-day dinner in an unventilated room. That it retards her progress is shown by her flushing afterwards. It is more important to have fresh air at meals, than at any other time. At Nordrach the windows are taken right out in the summer, and in winter are opened wide as soon as the patients are seated and served, thereby wonderfully assisting the appetite. My patient's bedroom at the English sanatorium was differently arranged from the one "M. O." describes. The bed was in the middle of the room, with a screen round the head, which was towards the window. This prevented any wind from blowing directly on the patient, the head particularly being protected, while the room was full of fresh air from the always open window. A fire was allowed when necessary to temper the air, and hot-water bottles were supplied, also in the beds in the verandah, and revolving huts, where the patients spent most of the daytime. The doctor regulated the amount of exercise, in some cases only a ten minutes' walk being allowed. One of the chief difficulties in private house treatment is the sitting out of doors where there is no verandah, and a hut cannot be afforded. My patient at present lies on a deck chair under a garden umbrella, a screen being erected by means of a clothes-horse, calico, and poles, fixed with rope and tent pegs, to keep the wind from blowing directly on her. I wish "M. O." all the success she deserves in her persevering efforts to regain health. It is most encouraging to hear, as we have done lately, of the numbers of people who do recover on the open-air system.

### Nursing in Tokio: A Difficulty.

By a Correspondent.

I, with another nurse, came out here last December as private nurses on a three years' engagement with the Colonial Nursing Association. I hold a four years' training certificate and also the L.O.S. certificate; my friend has a certificate for monthly nursing, as well as her general training certificate. We have also had good experience in nursing. We came to nurse Europeans or Americans, and have nothing to complain of as far as the agreement and work are concerned.

### The Registration of Midwives.

But my point is this. Since the new treaty came into force last summer the Japanese Government have complete power over foreigners residing in Japan. Now their law insists on the registration of midwives, and only those who have passed their examination for midwives can be registered, so that they made it compulsory for me (if I wished to be registered and practise as a midwife here) to go in for their examination. I have my certificates with me and they were translated into Japanese and submitted to the officials, but they might have been so much waste paper.

### The Examination.

I had to go through the examination without any preparation, which I really required, as it is three and a half years since I passed the L.O.S. examination. There were 90 Japanese entered as well as myself, but 60 only put in an appearance on the first day of the examination. It lasted five days in all, four days for the written part, and then, after an interval of twelve days, one day at the practical part. All the questions were in Japanese, and there was an interpreter to translate the questions into English for me. He was very closely watched, however, to see that he did no more than translate. The examination was about the same as that in England, a little more difficult perhaps. I did my best, but it certainly was annoying to be sent in unexpectedly and unprepared for an examination with Japanese, who have the name of being very clever. Quite to my surprise, I got through well, and not only well, but my interpreter wrote me afterwards the "best one." Out of the 60 only 18 were successful. The certificate I obtained will be a curiosity when I return to England, though it is all in Japanese, and I cannot tell whether I hold it right side up or not.

### Prospects of Nursing.

People out here are very kind to us, and we are getting on well. Not knowing the Japanese language is a great drawback, and it is difficult to learn. We live in a little Japanese house, and have a Japanese woman servant. The house is very nice and comfortable now, but in winter awfully cold. We have had plenty of work so far, and it looks as if the venture was going to be a success.

### Appointments.

Leek Memorial Cottage Hospital.—Mrs. Kathleen Buchanan has been appointed Matron. Miss Buchanan was trained at the North Staffordshire Infirmary and Eye Hospital, and afterwards held the post of charge nurse of the male surgical wards. She was then appointed sister of the female medical and gynæcological wards in the Royal Victoria Hospital, Belfast.

### Echoes from the Outside World.

### AN OPEN LETTER TO A HOSPITAL NURSE.

NEXT to the feeling of sorrow for the widowed Duchess of Saxe-Coburg is one of deep sympathy for our own beloved Queen that, in her old age, she should suffer so much pain and grief. Every one knows how often her heart has ached at the loss of life and the hardships and sickness entailed by the war in South Africa; how the anxious uncertainty which we have all been enduring with regard to our fellow country-men and women in China has been shared by Her Majesty; and now following fast upon the startling news of the assassination of the King of Italy we have the sad intelligence of the death of the Duke of Saxe-Coburg. Coming so soon after the death of his son, the event is the more distressing for his family, but for himself one can only be glad that a sudden attack of paralysis of the heart should have saved him from terrible suffering. For some time "Prince Alfred," as we used to call him, had been roaming from one watering place to another in search of health, which always eluded his grasp, and lately he had been subject to attacks of suffocation owing to a cancerous growth in the tongue and throat. The successor to the throne, you will remember, is the little Duke of Albany, who is only just 16, and who is living and being educated in Coburg so as to grow in touch with the country of his adoption. Until he attains his majority the Hereditary Prince of Hohenlohe-Langenburg will be his guardian and act as Regent.

THE horror with which the tidings of the assassination of the King of Italy was received on Monday was intensified because we all felt how narrowly a few weeks back the Prince of Wales had escaped the same fate. It is said, and I fear with much truth, that the fact of the cowardly Belgian court having released Sipido unpunished had so encouraged the Anarchists that they had developed fresh energy on every side. Certainly the Anarchist papers have latterly been strongly urging regicide upon their readers, with the sad result that a monarch of whom all had golden opinions has been cruelly murdered. As a nation we have always been particularly friendly with Italy, and reports of the kindly and lovable deeds of Queen Margaret and her husband have frequently reached us. I remember at the time when Italy was visited by a severe epidemic how both the King and Queen went fearlessly in and out of the wards of the hospital, by their sympathy helping the suffering, and by their bravery allaying the fears of the panic-stricken. Strangely enough, an hour or two before the tragedy was reported I had been admiring a picture of the Queen of Italy in a contemporary, clad as colonel of a German regiment, and thinking how handsome and happy she looked. And now, poor woman, she is suddenly called upon to bear almost the greatest grief a woman can know.

THE intelligence that General Prinsloo and a large number of Boers have surrendered unconditionally is extremely important. Lately the war in South Africa has appeared to "drag its slow length along," and though one has no doubt that the delay is unavoidable, every fresh recognition by the enemy of the inevitable end is most welcome. To General Hunter's brilliant strategy this capitulation is largely due. So far as the Legations at Peking are concerned, the message supposed to emanate from Sir Claude MacDonald, received in London on Monday night by the Admiralty, and dated July 21st, is the first ray of light which has been shed on the situation. It does not, of course, prove any more than the message from Mr. Conger of the same date that the peril is over, but, at any rate, it makes one hopeful that the massacres did not take place at the time reported. Meanwhile, Li Hung Chang makes the sinister proposal that the European Ministers and their families should be treated as hostages, to be sacrificed in the event of an advance upon the Chinese capital.

I WENT on Saturday morning to see Lady Randolph Churchill converted into Mrs. George Cornwallis West. The prominent position she has taken in connection with the "Maine" has evidently made her a special object of interest in the nursing world, for amongst the great crowd outside St. Paul's, Knightsbridge, I saw a considerable number of wearers of nurses' bonnets and veils. The church was most tastefully decorated with palms, white lilies, and scarlet gladioli. I do not know whether the choice of the latter to relieve the mass of white was Lady Randolph's own, or whether it was all arranged by the florist, but the glowing, vivid blossoms seemed to me singularly appropriate to the striking handsomeness of the bride. I have never seen her look so well or so happy, and—may I say it?—so wonderfully young. Her dress was of cloudy blue chiffon, which "swam" around her as she walked, the bottom of the skirt being edged with a deep flounce of beautiful Cluny lace of a full shade of cream. A bolero of the same lace was on the bodice, and the toque was a cunning combination of chiffon and lace and cream roses. Instead of the regulation shower bouquet, Lady Randolph carried just a handful of less than a dozen white roses, tied loosely together as if they had just been gathered from a garden. The Duke of Marlborough, who looked very small by the side of his kinswoman, gave the bride away, and made a strong contrast to her escort when she returned from the ceremony, for Mr. George Cornwallis West is very tall, quite a head taller than his wife, and he is a fine, well set up young fellow, looking far older than his years. The ovation which was given to the happy pair as they stepped into their carriage must have been a source of great pleasure to the recipients. I think few of us had realised how deeply Lady Randolph and her colleagues' efforts to alleviate the suffering of the soldiers had won the hearts of the multitude. "May you live long," "Health and happiness to Lady Randolph," and similar good wishes were forthcoming from many women of the working classes who crowded around the windows of the carriage and almost stopped the horses. Amongst the guests I specially noticed Lady Sarah Wilson, who had just arrived from Mafeking. She is a good deal like her sister, Lady Georgiana Curzon, only thinner and taller. To see her thus, a guest at a fashionable wedding, it was hard to realise the experiences she had so lately undergone in her bomb-proof shelter.

IT has always been a fable that in August London is empty, that there is nothing to be done, and no one to be seen. So seriously was this once believed, that a fashionable person having to remain in town after July would hardly dare show herself lest she should at once be classed as a "nobody." It is a striking commentary upon these ridiculous ideas that not only is the Shah of Persia coming to Buckingham Palace in what is called the "dead month," but the Prince of Wales and the Duke of York, as well as the Lord Mayor of London and the civic body, will also be in the capital just then. Next Wednesday about midday the Shah will arrive at Charing Cross Station (I specially mention the time lest some of you might like to arrange for a peep at the dusky potentate in your off-duty leisure), then he will go to Osborne, to the Crystal Palace, to Virginia Water, to Aldershot, to Portsmouth, to Manchester, and to Brighton. All this means plenty of opportunity for everyone to see him who wishes to do so. He leaves Dover again on August 21st.

### For Reading to the Sick.

"SURELY He hath borne our griefs."

Christ's heart was wrung for me, if mine is sore;
And if my feet are weary, His have bled;
He had no place wherein to lay His head.
If I am burdened, He was burdened more,
He felt the unuttered anguish which I dread;
He hungered, who the thousands hungry fed,
And thirsted, who the world's refreshment bore.

If grief be such a looking-glass as shows
Christ's face and man's in some sort made alike,
Then grief is pleasure with a subtle taste.
Wherefore should it fret, or faint, or haste?
Grief is not grievous to a soul that knows
Christ comes—and listens for that hour to strike.

—*Christina Rossetti.*

**Reading.**

To those who have any true knowledge of God, and faith in Him as a Father, the consciousness that all their afflictions come from Him is the chief of comforts. Is it not far easier to bear what we believe He orders, than that which seems to come merely from man, or by chance? We have noticed already that this feeling often finds expression. David gave utterance to it when, in making choice of various punishments offered for his acceptance, he said (2 Sam. xxiv. 14), "Let us fall now into the hand of the Lord; for His mercies are great: and let me not fall into the hand of man."

The higher our faith rises, the more clearly it is able to perceive that all distresses alike come from God, from the same great First Cause, though some by more direct channels, the less difficult will it be to endure patiently.

That Christ was smitten of God concerns ourselves. Whatever the knowledge of it may have been to Him, in whatever way it affected His human mind and spirit, it is certainly one of the utmost importance to us.

Because it is the assurance of a purpose in His sorrows.

Since they were not by chance, no mere blast of ill fortune, nor of the will of man, but from God, they must have been deliberate, and have had a definite object and end. And the purpose of God cannot fail. And was not the object our salvation?

If that Christ was a Man of Sorrows and acquainted with grief came from His Father, all grief and sorrow must be capable of explanation, as coming from God or allowed by Him. Any apparent inconsistency will be reconciled by-and-by. For no problem of suffering could be harder to solve, in itself, than why Christ should have been so afflicted.

Shall we not hear the pleading voice of the Man of Sorrows crying, as though herein lay the very extremity of His acquaintance with grief, "Have pity upon Me, have pity upon Me, O my friends; for the hand of God hath touched Me"?—*W. St. Hill Bourne.*

Spake my heart by sorrow smitten,
"Where, O where is comfort found?"
Earth is barren, waste and weary,
Barren as the desert ground;
Aching hearts can find no comfort,
Comfort ne'er on earth was found.

In the silence of my sorrow,
Came a voice alluring, sad,
"Seek the hand that gives the sorrow,
There is comfort to be had,"—
Strange, that in the hand that wounds me
Is my comfort to be had!

Sought I for the Hand that smote me,
Ah! 'twas wounded very sore,
But His soul was pure as sunlight,
And I felt my grief no more;
He Who spake the word of comfort,
He was wounded very sore.

### Notes and Queries.

THE Editor is always willing to answer in this column, without any fee, all reasonable questions, as soon as possible.

But the following rules must be carefully observed:—

1. Every communication must be accompanied by the name and address of the writer.
2. The question must always bear upon nursing, directly or indirectly.

If an answer is required by letter a fee of half-a-crown must be enclosed with the note containing the inquiry.

*Sanitary Inspection.*

(171) Would you kindly tell me how to become a lady sanitary inspector, or to whom to apply for information on the subject?—*M. C.*

Miss Lamport, 52, St. John's Wood Road, N.W., would supply you with all particulars, or you might apply to the Principal of Bedford College, or the Secretary of the National Health Society, 53, Berners Street, W.

*Uniform.*

(172) Will you kindly inform me if the uniform for the Army Reserve Sisters sent out to South Africa is the same as those worn at Netley, and what the climate is like at the present time?—*Inquirer.*

1. Not exactly. 2. Exceedingly hot during the day and bitterly cold at night.

*Oxygen Home.*

(173) I shall be greatly obliged if you will give me the address of the Oxygen Home in London.—*Q. V. J. I.*

Fitzroy Square, W.C.

*Over Forty.*

(174) Having read the question put by "Over Forty," I beg to say that your correspondent could be admitted at Salisbury General Infirmary, where excellent training is given. As paying probationer her age would not be objected to. Entrance fee is £5 5s. for six months. Rooms would have to be found in the town. I could give addresses if required.—*Nurse P.*

*Cancer.*

(175) Will you kindly tell me if it is usual to "mass" cancerous people and if it is done in the Cancer Hospital; also if it is dangerous to the masseuse?—*Nina Caldwell.*

So far as present experience goes there seems no danger to the masseuse.

*Dispensing.*

(176) Would you kindly let me know what is the preparation required in order to obtain the Local Government Board certificate for dispensing, where and when the exams. are held, and the best way of preparing for them?—*Jno. L.*

The Local Government Board do not give certificates for dispensing. Apply for particulars to the Secretary of the Pharmaceutical Society, Bloomsbury Square, W.C., and the Secretary of the Society of Apothecaries, Blackfriars, E.C.

*Children's Ailments.*

(177) Can you tell me of any good handbook on children's ailments, with their symptoms and simple remedies, for a young mother going out to Ceylon? It must not be large, and in quite simple language.—*L. C.*

"Chevasse's Advice to a Mother on the Management of Her Children." (J. and A. Churchill). 2s. 6d.

*Convalescent Home.*

(178) Will you kindly let me know the addresses of some "seaside homes" for consumptives, or other invalids. The charges must be moderate.—*Inquirer.*

You do not state sex or locality desired. These are general homes: Royal West of England Sanatorium, Weston-super-Mare; with subscriber's note, 5s. a week, without 18s. Seaford Seaside Convalescent Hospital; 5s. a week with subscriber's letter. Convalescent Home, Fairfield House, Lowestoft; with subscriber's letter, 4s. St. Andrew's Convalescent Home, East Cliff, Folkestone; with subscriber's letter, 2s. 6d. a week for three weeks, otherwise 10s. 6d. weekly.

*Monthly Nurse.*

(179) A lady wrote to me in May asking if I could take her case from August 1st. I wrote back, stating fee, &c., heard nothing for a fortnight, and wrote again asking for a decision. I still had no reply, and have lost another case through waiting.—*H. S.*

It is better in such cases not to wait for anyone.

*South Africa.*

(180) A nurse going to South Africa for service at the front would like to know some particulars with regard to outfit and general requirements. —*Louise.*

You will find all you want to know in an article, "The Princess of Wales' Nurses on their Way to South Africa," in THE HOSPITAL Nursing Mirror of March 10th.

**Standard Books of Reference.**

"The Nursing Profession: How and Where to Train." 2s. net; post free 2s. 4d.

"The Nurses' Dictionary of Medical Terms." 2s.

"Burdett's Series of Nursing Text-Books." 1s. each.

"A Handbook for Nurses." (Illustrated.) 5s.

"Nursing: Its Theory and Practice." New Edition. 3s. 6d.

"Helps in Sickness and to Health." Fifteenth Thousand. 5s.

All these are published by THE SCIENTIFIC PRESS, Ltd., and may be obtained through any bookseller or direct from the publishers, 28 & 29, Southampton Street, London, W.C.

### Travel Notes.

LIV.—ANDERNACH AND ITS SURROUNDINGS.

You will find by referring to the "Nursing Mirror" for July 22nd last, that I gave an account of Konigswinter and the country round about. To-day I shall give you some account of Andernach, which is the next best centre between Bonn and Coblentz. Of this latter place I wrote an article on September 23rd, so that with the account of Andernach to-day, we shall have studied the tourist capabilities of the Rhine from Cologne to beyond Coblentz fairly exhaustively.

THE JOURNEY.

The expense to Cologne I gave you last week—to Bonn, if you go all the way in the Netherlands steampship boats, will be £1 15s. 10d. first class return, and £1 5s. 1d. second return. If you elect to go by Harwich, it will be first-class return £3 11s. 6d., and second return £2 7s. 9d., very nearly twice the amount you see; however, delightful as the Netherlands steamship route is, it has the disadvantage of taking time which cannot always be spared.

ACCOMMODATION AT ANDERNACH.

Living is cheap in modest Andernach, and surely that is a great recommendation to most of us. The Rheinischer Hof treats you handsomely for four marks a day, and for a prolonged stay probably their terms would be less. It is not a place in which the most extravagantly disposed *can* spend lavishly, for the excellent reason that there is nothing on which to spend. Almost all the excursions can be done on one's feet, or by the rail and steamer; even the beautiful valley of the Ahr excursion necessitates no heavy expenditure.

ATTRACTIONS OF ANDERNACH.

There are none in the usual acceptation of the word, no casino or kurhaus, thank heaven, no promenade, no band, nothing to attract the gay world, hence the sweet simplicity of cheapness still lingers in its old walls. It is one of the most picturesque towns on the Rhine, and as yet but little known to tourists. I feel almost guilty in giving it even this modest publicity. Artists will find it a charming retreat, full of interest and of subjects for the brush or pencil. It retains a good portion of its old walls and narrow crooked streets, and has not bowed before the hand of the "tasteful" restorer and hygienic meddler like Boppard, once so entrancing and now so prosaic. The effect as you approach from the river is very picturesque; first one remarks the watch tower in two tiers, round below and octagonal above; the moment you see it, you recognise an old friend of your youth, frequently depicted in the horrible drawing books, with the assistance of which, in the schoolroom we unwillingly attempted the thorny paths of art. This dates from 1451, and looks equal to another four or five hundred years of exposure to time and the elements. Then there is the Rathhaus, very little altered from its original building in 1564; the outside is far more interesting than the interior, full as usual of weariful Roman antiquities, the very nature of which, after much wandering in many lands, caused me to feel an approaching headache and general *malaise*. I fly before them as from a pestilence. More interesting, however, is a remarkable cistern of prodigious and awesome depth, called the "Jew's bath." I have not been able to learn a reliable explanation of this name. It seems to me to hint darkly at wholesale murder of the unhappy Israelites. There is but little left of the Castle of the Electors; as usual, the French in their destructive spirit almost levelled it with the ground in 1688. The parish church will delight you, I know, if your tastes lie that way. I was first inspired to visit Andernach by seeing a small picture of its interior in twilight. I endeavoured to emulate that successful artist when I was there, result—lamentable failure. Try to see it for the first time in the gloaming, the ideal moment is on an autumn late afternoon, when the few lights are not yet extinguished after a service.

EXCURSIONS—THE AHR VALLEY.

I think I had better first tell of the Ahr Valley, lest I should leave myself scanty space for its charms. You must make an early start, and take the first train (or cycle) to Remagen, which is the starting point for the railway ascent of the Ahr valley. Fares: 1st return to Adenau, 3 marks 40; 2nd, 2 marks 60; and 3rd, 1 mark 70. The distance is 26 miles, and the time occupied 2¼ hours. I must not say much about the beauties of the route, I should not know where to stop. At Heimersheim there is a pleasant excursion to be made to Burg Neuenahr, that can be managed any morning, for Heimersheim is the first station up the valley, only five miles from Remagen. Neuenahr is by no means a striking place, only it gives an excuse for exploring the country; unless one has an object, one's wanderings are often so aimless and resultless. Ahrweiler is another good halting place for a separate excursion, and is more individually interesting than Neuenahr. At every station you may alight and explore fresh ground with much delight, but on the first occasion you had better go the whole journey.

TO THE LAACHER SEE.

There are two ways to do this. Railway from Andernach to Niedermendig, and then walk on the Laacher See; you must allow an hour for the walk, A second way is by train to Kruft, and then walk. This involves much more walking, but is rather a prettier road, perhaps, on the whole. It is a good plan to go one way and return the other. The quarries at Niedermendig are worth seeing. I was informed (which indeed was no more than I expected) that those wary old Romans had worked them! I feel it would be such a treat to get the better of those old fellows somehow, but one never can. A third way, better than all, if you can stand a very long walk, is to take train to Brohl, and then walk to the Laacher See, or take a carriage if you like (five marks, quite worth it), returning by Niedermendig or Kruft. Another charming walk is to ascend the valley on foot as far as you feel inclined, behind Sinzig. Sinzig has a very fine church, and still part of its ancient walls.

### Minor Appointments.

ASTON WORKHOUSE INFIRMARY, GRAVELLY HILL.—Miss Kate Mary Cutler has been appointed Charge Nurse. She was trained at Mill Road Infirmary, Liverpool, and has since been charge nurse successively at Prescot Union Infirmary, Sheffield Union Infirmary, and St. George's Infirmary, Fulham Road, London.

DOUGLAS ISOLATION HOSPITAL, DOUGLAS, ISLE OF MAN.—Miss Hilda Davison has been appointed Nurse in Charge. She was trained at the City of Glasgow Fever Hospital, Knightswood, where she has been charge nurse. She has also been sister in charge of enteric pavilions, Middle Wood Hospital, Motherwell.

BLABY AND WIGSTON MAGNA JOINT HOSPITAL, LEICESTER.—Miss Teresa Hayer has been appointed Assistant Nurse. She was trained at St. Olave's Infirmary, Rotherhithe, London.

NEWPORT AND MONMOUTHSHIRE HOSPITAL.—Miss E. Thompson has been appointed Charge Nurse. She was trained at Leeds General Infirmary.

### Resignation.

MISS PEARSON, nurse of the children's ward in connexion with the West Cornwall Women's Hospital at Redruth, has resigned her appointment as nurse in consequence, it is stated, of the indisposition of her mother, Mrs. Pearson, of Coombe Vicarage.

## Figures and Tables

**Fig. 21. f1:**
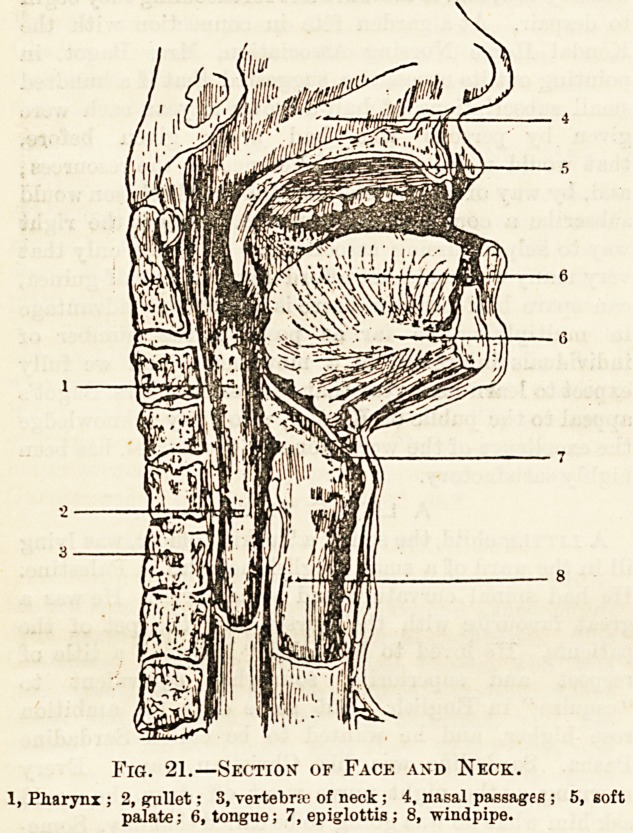


**Fig. 22. f2:**